# Supplementary material for: Leptin and PAI-1 Levels Are Decreased After a Dietary Intervention in Patients with Irritable Bowel Syndrome
Source: Int J Mol Sci. 2025 May 11;26(10):4607. doi: 10.3390/ijms26104607 (PMC12111115; doi:10.3390/ijms26104607)
Supplement: Supplementary file 1 [file ijms-26-04607-s001.zip › ijms-3524181-supplementary.pdf]

Supplementary Table S1. IBS subgroups at baseline, 4 weeks, and 6 months

	SSRD<br>N = 70	Low FODMAP<br>N = 72	P-value
**Baseline (n, %)**			0.157
IBS-C	13 (18.6)	12 (16.7)	
IBS-D	27 (38.6)	15 (20.8)	
IBS-M	19 (27.1)	29 (40.3)	
IBS-U	2 (2.9)	4 (5.6)	
FBD	9 (12.9)	12 (16.7)	
**4 weeks (n, %)**			0.828
IBS-C	8 (11.4)	10 (13.9)	
IBS-D	8 (11.4)	7 (9.7)	
IBS-M	12 (17.1)	9 (12.5)	
IBS-U	0	2 (2.8)	
FBD	23 (32.9)	25 (34.7)	
Healthy	19 (27.1)	19 (26.4)	
**6 months (n, %)**			0.244
Missing	19 (27.1)	23 (31.9)	
IBS-C	6 (8.6)	11 (15.3)	
IBS-D	8 (11.4)	6 (8.3)	
IBS-M	11 (15.7)	9 (12.5)	
IBS-U	1 (1.4)	3 (4.2)	
FBD	22 (31.4)	13 (18.1)	
Healthy	3 (4.3)	7 (9.7)	

SSRD = starch- and sucrose-reduced diet. Low FODMAP = low content of fermentable oligo-, di-, and monosaccharides and polyols. IBS-C = constipation-predominated IBS, IBS-D = diarrhea-predominated IBS, IBS-M = mixed IBS, IBS-U = unspecified IBS, and FBD = unspecified functional bowel disorder according to Rome IV questionnaire [49]. Healthy was defined as total IBS-SSS score <75 [50]. FBD at baseline represented those with abdominal pain but with weak association to bowel habits. At 4 weeks and 6 months, FBD represent those with abdominal pain 2-3 times or less per month but with a total IBS-SSS score >75. Values are given as numbers and percentages. Fisher's exact test. $P < 0.05$ was considered statistically significant.

Supplementary Table S2. Previous or present comorbidity and drug treatments

**Comorbidity**	**IBS**<br>**N=142**
Allergy	16 (11.3)
Anxiety	4 (2.8)
Asthma bronchialis	6 (4.2)
Burned out	7 (4.9)
Depression	10 (7.0)
Eczema	17 (12.0)
Fibromyalgia	8 (5.6)
Hypertension	11 (7.7)
Hypothyroid disease	6 (4.2)
Lactose intolerance	9 (6.3)
Migraine/headache	9 (6.3)
Reflux/hiatus hernia	17 (12.0)
**Drug treatment**	
Allergy medicines	22 (15.5)
Antidepressants	22 (15.5)
Asthma inhalators	6 (4.2)
Hormonal treatment*	22 (15.5)
Laxatives/bulking agents	34 (23.9)
Levaxine	6 (4.2)
NSAID	44 (31.0)
Paracetamols	50 (35.2)
Proton pump inhibitors	43 (30.3)
**Dietary supplements**	
Iron	7 (4.9)
Other minerals (Ca, Mg, Z, Si)	20 (14.1)
Vitamin B/Folic acid	10 (7.0)
Vitamin D	23 (16.2)
Multivitamins	21 (14.8)
Vitamin D and/or multivitamins	36 (25.4)
Probiotics	13 (9.2)

IBS = irritable bowel syndrome. *= combination pills. Values are given as number (percentage).

Supplementary Table S3. Weight and nutrient intake at baseline, 4 weeks, and 6 months

	SSRD, N=70		Low FODMAP, N=72		P-value§
	**Median (IQR)**	**P-value**	**Median (IQR)**	**P-value**	
**Weight (kg)**					
Baseline	71.3 (63.9-82.7)	-	68.8 (63.2-83.2)	-	0.582
4 weeks	70.1 (63.2-80.9)	<0.001	67.6 (62.3-82.7)	<0.001	0.792
6 months<br>*Missing;*	74.1 (66.4-85.6)<br>19	0.678	68.6 (62.9-80.8)<br>23	0.078	0.131
**Kilocalorie intake (kcal)**					
Baseline<br>*Missing*	1792.2 ± 536.2	-	1812.2 ± 502.2<br>2	-	0.820
4 weeks<br>*Missing*	1520.8 ± 475.1<br>4	<0.001	1667.1 ± 499.6<br>4	<0.001	0.085
6 months<br>*Missing*	1791.2 ± 613.0<br>26	0.012	1675.7 ± 739.1<br>25	0.007	0.421
**Carbohydrate (g)**					
Baseline<br>*Missing*	176.5 ± 61.3	-	181.7 ± 51.8<br>2	-	0.590
4 weeks<br>*Missing*	89 ± 37<br>4	<0.001	155 ± 54<br>4	<0.001	<0.001
6 months<br>*Missing*	155.2 ± 63.8<br>26	0.038	159.9 ± 69.6<br>25	0.014	0.740
**Protein (g)**					
Baseline<br>*Missing*	70.2 (52.5-80.6)	-	66.4 (52.2-79.0)<br>2	-	0.614
4 weeks<br>*Missing*	78.9 (59.6-94.9)<br>4	0.003	67.5 (51.4-81.2)<br>4	0.467	0.017
6 months<br>*Missing*	75.9 (55.1-86.5)<br>26	0.797	66.2 (48.9-81.0)<br>25	0.597	0.133
**Fat (g)**					
Baseline<br>*Missing*	75.2 (59.4-93.1)	-	75.9 (55.4-96.2)<br>2	-	0.987
4 weeks<br>*Missing*	82.7 (65.3-108.3)<br>4	0.212	76.4 (52.2-93.1)<br>4	0.308	0.060
6 months<br>*Missing*	90.7 (51.3-112.1)<br>26	0.894	71.2 (48.2-89.4)<br>25	0.071	0.080
**Fiber (g)**					
Baseline<br>*Missing*	17.6 (12.4-23.7)	-	17.9 (15.1-22.1)	-	0.535
4 weeks<br>*Missing*	16.0 (12.2-22.0)<br>4	0.134	15.2 (11.3-20.4)<br>4	0.002	0.735
6 months<br>*Missing*	17.6 (12.7-23.5)<br>26	0.513	14.9 (10.0-20.0)<br>25	<0.001	0.155
**Monosaccharide intake (g)**					
Baseline<br>*Missing*	27.8 (19.4-39.4)	-	27.3 (18.5-34.4)<br>2	-	0.594
4 weeks	17.2 (10.2-34.5)	<0.001	21.2 (15.1-28.0)	0.002	0.405

Missing	4		4		
6 months	28.6 (15.1-35.0)	0.176	24.5 (15.0-35.2)	0.216	0.577
Missing	26				
**Disaccharide (g)**					
Baseline	36.3 (22.7-53.8)	-	41.0 (28.0-55.7)	-	0.280
Missing			2		
4 weeks	15.6 (11.9-27.1)	<0.001	31.2 (23.5-49.2)	0.118	<0.001
Missing	4		4		
6 months	26.9 (18.7-43.9)	0.027	29.6 (18.5-53.4)	0.105	0.600
Missing	26		25		
**Sucrose (g)**					
Baseline	24.7 (14.0-41.4)	-	25.5 (15.2-44.1)	-	0.433
Missing			2		
4 weeks	6.2 (2.9-9.6)	<0.001	17.9 (12.5-35.6)	0.027	<0.001
Missing	4		4		
6 months	16.5 (10.5-30.9)	0.013	22.2 (10.3-35.9)	0.079	0.432
Missing	26		25		
**Starch intake (g)**					
Baseline	43.8 ±22.4	-	52.0 ±25.2	-	0.045
Missing			2		
4 weeks	19.8 ± 20.1	<0.001	48.9 ± 25.0	0.515	<0.001
Missing	4		4		
6 months	40.1 ± 24.4	0.220	41.6 ± 27.7	0.008	0.783
Missing	26		25		
**Added sugar (g)**					
Baseline	33.3 (15.9-46.5)	-	31.2 (16.7-49.4)	-	0.973
Missing			2		
4 weeks	4.6 (1.5-10.5)	<0.001	24.1 (12.8-42.5)	0.134	<0.001
Missing	4		4		
6 months	16.9 (7.8-38.0)	0.024	24.0 (8.9-52.8)	0.472	0.270
Missing	26		25		

SSRD = starch- and sucrose-reduced diet. Low FODMAP = low content of fermentable oligo-, di-, and monosaccharides and polyols. Values are given as median (interquartile ranges (IQR)) or mean and ± standard deviation. Wilcoxon Signed Ranks or paired samples T-test for comparison within the groups and Mann-Whitney U test or independent sample T-test for comparison between the two groups (§). $P < 0.05$ was considered statistically significant.

Supplementary Table S4. Gastrointestinal symptom scoring

	SSRD N=70		Low FODMAP N=72		P-value§
**VAS-IBS**	**Median (IQR)**	**P-value**	**Median (IQR)**	**P-value**	
**Abdominal pain** 5 (1-13)					
Baseline<br>*Missing*	47 (29-64)	-	50 (33-65)<br>1	-	0.431
4 weeks<br>*Missing*	16 (0-31)	<0.001	13 (0-27)<br>1	<0.001	0.502
6 months<br>*Missing*	34 (18-64)<br>18	0.012	30 (16-54)<br>23	<0.001	0.683
**Diarrhea** 3 (0-10)					
Baseline<br>*Missing*	52 (18-73)	-	36 (2-73)<br>1	-	0.201
4 weeks<br>*Missing*	16 (3-37)	<0.001	8 (0-25)<br>1	<0.001	0.024
6 months<br>*Missing*	33 (8-69)<br>18	0.002	11 (3-44)<br>23	0.009	0.023
**Constipation** 6 (2-16)					
Baseline<br>*Missing*	52 (7-71)	-	50 (10-75)<br>1	-	0.635
4 weeks<br>*Missing*	15 (2-42)	<0.001	21 (0-55)<br>1	<0.001	0.635
6 months<br>*Missing*	21 (0-62)<br>18	0.180	42 (3-70)<br>23	0.023	0.285
**Bloating and flatulence** 10 (2-23)					
Baseline<br>*Missing*	73 (59-89)		75 (59-86)<br>2		0.817
4 weeks<br>*Missing*	25 (10-54)	<0.001	19 (8-50)<br>2	<0.001	0.150
6 months<br>*Missing*	62 (30-76)<br>18	0.005	56 (33-70)<br>23	<0.001	0.523
**Vomiting and nausea** 2 (0-4)					
Baseline<br>*Missing*	13 (3-35)		13 (1-37)<br>1		0.716
4 weeks<br>*Missing*	3 (0-12)<br>1	<0.001	0 (0-11)<br>1	<0.001	0.370
6 months<br>*Missing*	7 (0-21)<br>18	0.004	5 (0-21)<br>23	0.010	0.465
**Intestinal symptoms' influence on daily life** 2 (0-14)					
Baseline<br>*Missing*	75 (59-85)	-	70 (55-80)<br>1	-	0.572
4 weeks	24 (12-63)	<0.001	22 (10-50)	<0.001	0.385

Missing			1		
6 months	40 (23-77)	<0.001	48 (24-69)	<0.001	0.625
Missing	18		23		
**Psychological well-being** 5 (2-15)					
Baseline	40 (15-65)	-	35 (13-57)	-	0.393
Missing			1		
4 weeks	20 (5-33)	<0.001	18 (2-34)	<0.001	0.696
Missing			1		
6 months	22 (10-50)	0.007	26 (8-39)	0.043	0.739
Missing	18		23		
**Total IBS-SSS**					
Baseline	306 (231-349)	-	307 (238-357)	-	0.880
Missing			1		
4 weeks	119 (63-226)	<0.001	116 (63-176)	<0.001	0.474
Missing			1		
6 months	204 (150-326)	<0.001	220 (144-301)	<0.001	0.327
Missing	19		22		

SSRD = starch- and sucrose-reduced diet. Low FODMAP = low content of fermentable oligo-, di-, and monosaccharides and polyols. Symptoms measured by irritable bowel syndrome-severity scoring system (IBS-SSS) [50] and Visual Analog Scale for IBS (VAS-IBS) [51], with reference values within brackets [61]. Values are given as median (interquartile ranges (IQR)) or mean and ± standard deviation. Wilcoxon Signed Ranks for comparison within the groups and Mann-Whitney U test for comparison between the two groups (§). $P < 0.05$ was considered statistically significant.

Supplementary Table S5. Extraintestinal symptom scoring

	SSRD N=70		Low FODMAP N=72		P-value§
**Extraintestinal IBS-SSS**	**Median (IQR)**	**P-value**	**Median (IQR)**	**P-value**	
**Difficulties eating a whole meal**					
Baseline<br>*Missing*	10 (2-25)	-	6 (0-20)<br>1	-	0.241
4 weeks<br>*Missing*	2 (0-12)	<0.001	0 (0-9)<br>1	<0.001	0.206
6 months<br>*Missing*	4 (0-12)<br>19	0.061	3 (0-18)<br>22	0.053	0.800
**Headache**					
Baseline<br>*Missing*	34 (7-66)	-	28 (9-58)<br>2	-	0.869
4 weeks<br>*Missing*	14 (2-32)	<0.001	12 (0-35)<br>1	<0.001	0.759
6 months<br>*Missing*	24 (7-55)<br>19	0.158	20 (4-50)<br>22	0.002	0.454
**Back pain**					
Baseline<br>*Missing*	20 (5-50)	-	25 (2-62)<br>1	-	0.656
4 weeks<br>*Missing*	5 (0-28)	<0.001	4 (0-35)<br>1	<0.001	0.716
6 months<br>*Missing*	24 (4-60)<br>19	0.242	24 (4-70)<br>22	0.670	0.713
**Fatigue**					
Baseline<br>*Missing*	59 (33-83)	-	73 (47-83)<br>1	-	0.199
4 weeks<br>*Missing*	28 (10-56)	<0.001	37 (14-60)<br>1	<0.001	0.470
6 months<br>*Missing*	50 (18-68)<br>19	0.007	48 (19-69)<br>22	<0.001	0.564
**Belching/excess wind**					
Baseline<br>*Missing*	74 (50-85)	-	75 (54-87)<br>1	-	0.686
4 weeks<br>*Missing*	14 (7-40)	<0.001	21 (8-45)<br>1	<0.001	0.473
6 months<br>*Missing*	47 (23-69)<br>19	<0.001	48 (22-70)<br>22	<0.001	0.817
**Reflux**					
Baseline<br>*Missing*	22 (9-49)	-	19 (0-62)<br>1	-	0.376
4 weeks<br>*Missing*	5 (0-21)	<0.001	3 (0-26)<br>1	<0.001	0.965
6 months<br>*Missing*	11 (5-28)<br>19	0.003	21 (2-54)<br>22	0.014	0.456
**Urinary urgency**					
Baseline	14 (3-65)	-	21 (4-63)	-	0.638

Missing			1		
4 weeks	4 (0-20)	<0.001	3 (0-22)	<0.001	0.899
Missing			1		
6 months	19 (0-45)	0.008	16 (0-53)	0.004	0.962
Missing	19		22		
**Leg pain**					
Baseline	1 (0-8)	-	0 (0-12)	-	0.977
Missing			1		
4 weeks	0 (0-6)	0.037	0 (0-5)	0.005	0.668
Missing			1		
6 months	2 (0-10)	0.870	0 (0-14)	0.564	0.420
Missing	19		22		
**Muscle/joint pain**					
Baseline	23 (5-55)	-	29 (3-72)	-	0.619
Missing			1		
4 weeks	13 (0-33)	<0.001	12 (0-39)	<0.001	0.650
Missing	1		1		
6 months	23 (5-54)	0.068	19 (4-70)	0.083	0.538
Missing	19		22		
**Total extraintestinal IBS-SSS**					
Baseline	11 (110-206)	-	168 (119-241)	-	0.455
Missing			2		
4 weeks	91 (27-145)	<0.001	77 (45-136)	<0.001	0.949
Missing	1		1		
6 months	127 (74-192)	<0.001	133 (78-214)	<0.001	0.654
Missing	19		22		

SSRD = starch- and sucrose-reduced diet. Low FODMAP = low content of fermentable oligo-, di-, and monosaccharides and polyols. Symptoms measured by irritable bowel syndrome-severity scoring system (IBS-SSS) [50]. Values are given as median (interquartile ranges (IQR)) or mean and ± standard deviation. Wilcoxon Signed Ranks for comparison within the groups and Mann-Whitney U test for comparison between the two groups (§). P < 0.05 was considered statistically significant.

Supplementary Table S6. Association of change in PAI-1 (dependent variable) with change in weight and nutrient intakes

Independent variable	β-coefficient and 95% CI	P-value
Weight (kg)	0.134 (-3.374-3.641)	0.941
Kilocalorie intake (kcal)	0.006 (-0.002-0.015)	0.140
Carbohydrate intake (g)	0.019 (-0.053-0.091)	0.606
Protein intake (g)	0.115 (-0.080-0.310)	0.248
Fat intake (g)	0.094 (-0.033-0.221)	0.147
Fiber intake (g)	0.348 (-0.338-1.034)	0.320
Monosaccharide intake (g)	0.047 (-0.287-0.381)	0.783
Disaccharide intake (g)	0.011 (-0.159-0.181)	0.902
Sucrose intake (g)	0.021 (-0.186-0.229)	0.840
Starch intake (g)	-0.045 (-0.186-0.096)	0.527
Added sugar intake (g)	0.007 (-0.153-0.167)	0.931

PAI-1 = plasminogen activator inhibitor-1. Generalized linear model of association between changes in PAI concentration from baseline to 4 weeks as dependent variable and changes in single independent nutrient variables. Values are presented as β-values and 95% confidence interval. $P < 0.05$ was considered statistically significant.

Supplementary Table S7. Association of changes in PAI-1 (dependent variable) with change in nutrient intake, adjusted for change in weight, from baseline to 4 weeks

Adjusted independent variable	β-coefficient and 95% CI	P-value
Kilocalorie intake (kcal)	0.006 (-0.002-0.015)	0.147
Carbohydrate intake (g)	0.017 (-0.057-0.092)	0.647
Protein intake (g)	0.114 (-0.081-0.309)	0.252
Fat intake (g)	0.094 (-0.033-0.221)	0.145
Fiber intake (g)	0.348 (-0.337-1.034)	0.319
Monosaccharide intake (g)	0.053 (-0.283-0.389)	0.758
Disaccharide intake (g)	0.008 (-0.163-0.179)	0.929
Sucrose intake (g)	0.018 (-0.191-0.227)	0.866
Starch intake (g)	-0.051 (-0.195-0.092)	0.484
Added sugar intake (g)	0.003 (-0.159-0.165)	0.973

PAI-1 = plasminogen activator inhibitor-1. Generalized linear model of association between changes in PAI concentration from baseline to 4 weeks as dependent variable and changes in nutrient intake as independent variables adjusted for weight changes. Values are presented as β-coefficients and 95% confidence interval. $P < 0.05$ was considered statistically significant.

Supplementary Table S8. Association of change in symptoms with change in PAI-1, from baseline to 4 weeks

Symptom changes (dependent variable)	PAI-1 changes β-coefficient and 95% CI	P-values
Abdominal pain	0.109 (-0.074-0.293)	0.244
Diarrhea	0.069 (-0.076-0.214)	0.351
Constipation	-0.003 (-0.167-0.162)	0.976
Bloating and flatulence	0.118 (-0.047-0.282)	0.161
Vomiting and nausea	0.155 (-0.084-0.394)	0.203
Intestinal symptoms' influence on daily life	0.127 (-0.045-0.298)	0.148
Psychological well-being	-0.068 (-0.258-0.122)	0.482
Difficulties eating a whole meal	-0.019 (-0.261-0.224)	0.880
Headache	-0.026 (-0.233-0.181)	0.804
Back pain	-0.068 (-0.266-0.131)	0.505
Fatigue	0.127 (-0.072-0.326)	0.212
Belching/excess wind	0.178 (0.023-0.332)	0.025
Reflux	-0.012 (-0.197-0.173)	0.900
Urinary urgency	0.006 (-0.183-0.196)	0.946
Leg pain	0.064 (-0.208-0.336)	0.643
Muscle/joint pain	-0.028 (-0.225-0.170)	0.783
Total IBS-SSS	0.028 (-0.019-0.075)	0.239
Total extraintestinal IBS-SSS	0.029 (-0.047-0.105)	0.457

PAI-1 = plasminogen activator inhibitor-1. Symptoms measured by irritable bowel syndrome-severity scoring system (IBS-SSS) [50] and visual analog scale for irritable bowel syndrome (VAS-IBS) [51]. Generalized linear model of association between changes in symptom scores (dependent variable) from baseline to 4 weeks and changes in PAI (independent variable). Values are presented as β-coefficients and 95% confidence interval (CI). $P < 0.05$ was considered statistically significant.

Supplementary Table S9. Recommendations of fruit intake according to a starch- and sucrose-reduced diet

**Well tolerated**	**Tolerated by some**	**Not tolerated**
Avocado	Persimmons	Apples
Blackberries	Plums	Apricots
Blueberries	Raisins	Bananas
Boysenberries	Watermelon	Cantaloupe
Cherries		Dates
Cranberries		Grapefruit
Currants		Guava
Figs		Honeydew melon
Gooseberries		Mangos
Grapes		Nectarines
Kiwi		Oranges
Lemons		Peaches
Limes		Pineapple
Loganberries		Tangelos
Olives		Tangerines
Papaya		
Pears		
Pomegranates		
Prunes		
Raspberries		
Rhubarb		
Strawberries		

Supplementary Table S10. Recommendations of vegetable and legume intake according to a starch- and sucrose-reduced diet

**Well tolerated**	**Tolerated by some**	**Not tolerated**
Alfalfa sprouts	Edamame soybeans	Beets
Artichokes*	Jicamas	Black beans
Arugulas	Leeks	Black-eyed peas
Asparagus*	Okra	Butternut
Bamboo shoots	Pumpkin	Carrots
Bok choy	Snow peas	Cassava
Broccoli*	Tempeh	Chickpeas
Brussel sprouts*	Tofu	Corn
Cabbage*	Yellow wax beans	Garlic
Cauliflower*		Green peas
Celery		Lentils
Chard		Kidney beans
Chicories		Lima beans
Chives		Navy beans
Collard greens		Onion
Cress		Parsnips
Cucumber		Pinto beans
Eggplant		Potatoes
Endive		Soybeans
Green beans		Split peas
Kale		Sweet potatoes
Lettuce		Yams
Mung bean sprouts		
Mushrooms		
Mustard green		
Peppers		
Radishes		
Spaghetti squash		
Spinach		
Tomatoes		
Turnips		
Yellow squash		
Zucchini		

*Excess intake can cause bloating/flatulence in all individuals.

Supplementary Table S11. Validation of analysis for copeptin

Sample	Sample 1	Sample 2
Concentration (pmol/L)	5.0	102
Precision CV (%)	5	1.98
Between-day CV (%)	4.7	0.81

CV = coefficient of variance.

Supplementary Table S12. Validation of analysis for leptin

	Intra-assay Precision			Inter-assay Precision		
**Sample**	1	2	3	1	2	3
N	20	20	20	40	40	40
Mean (ng/mL)	64.5	146	621	65.7	146	581
SD (ng/mL)	2.14	4.32	20.0	3.56	6.17	20.6
CV (%)	3.3	3.0	3.2	5.4	4.2	3.5

CV = coefficient of variance. N = number. SD = standard deviation.

Supplementary Table S13. Validation of analysis for plasminogen activator inhibitor-1 (PAI-1)

	Intra-assay Precision		Inter-assay Precision	
**Sample**	1	2	3	4
N	12	12	6	6
Mean (ng/mL)	42.70	7.63	49.76	7.22
SD (ng/mL)	1.01	0.41	2.41	0.54
CV (%)	2.4	5.4	4.8	7.5

CV = coefficient of variance. N = number. SD = standard deviation.

Supplementary Table S14. Validation of analysis for C-peptide and insulin

Hormone	Level (nmol/L)	Imprecision (CV%)	Sample size
C-peptide	0.63 / 1.8	2.2 / 2.5	1120
Insulin	8 / 90	3.3 / 3.4	1020

CV = coefficient of variance.
